# Comparative Minimum Inhibitory and Mutant Prevention Drug Concentrations for Pradofloxacin and Seven Other Antimicrobial Agents Tested against Bovine Isolates of *Mannheimia haemolytica* and *Pasteurella multocida*

**DOI:** 10.3390/pathogens13050399

**Published:** 2024-05-09

**Authors:** Joseph M. Blondeau, Shantelle D. Fitch

**Affiliations:** 1 Department of Clinical Microbiology, Royal University Hospital and Saskatchewan Health Authority, Saskatoon, SK S7N 0W8, Canada; shantelle.fitch@saskhealthauthority.ca; 2 Department of Biochemistry, Microbiology and Immunology, Pathology and Laboratory Medicine and Ophthalmology, University of Saskatchewan, Saskatoon, SK S7N 0W8, Canada

**Keywords:** *Mannheimia haemolytica*, *Pasteurella multocida*, MIC/MPC, pradofloxacin

## Abstract

Pradofloxacin—a dual-targeting fluoroquinolone—is the most recent approved for use in food animals. Minimum inhibitory and mutant prevention concentration values were determined for pradofloxacin, ceftiofur, enrofloxacin, florfenicol, marbofloxacin, tildipirosin, tilmicosin, and tulathromycin. For *M. haemolytica* strains, MIC_50/90/100_ values were ≤0.016/≤0.016/≤0.016 and MPC_50/90/100_ values were 0.031/0.063/0.063; for *P. multocida* strains, the MIC_50/90/100_ values ≤0.016/≤0.016/0.031 and MPC_50/90/100_ ≤ 0.016/0.031/0.063 for pradofloxacin. The pradofloxacin C_max_/MIC_90_ and C_max_/MPC_90_ values for *M. haemolytica* and *P. multocida* strains, respectively, were 212.5 and 53.9 and 212.5 and 109.7. Similarly, AUC_24_/MIC_90_ and AUC_24_/MPC_90_ for *M. haemolytica* were 825 and 209.5, and for *P. multocida,* they were 825 and 425.8. Pradofloxacin would exceed the mutant selection window for >12–16 h. Pradofloxacin appears to have a low likelihood for resistance selection against key bovine respiratory disease bacterial pathogens based on low MIC and MPC values.

## 1. Introduction

Bovine respiratory disease (BRD), or shipping fever, is a complex multi-factorial syndrome in cattle precipitated by viral infection and/or a variety of known stressors (weather, transport, co-mingling, castration, dehorning, weaning, and auction), which, in turn, predispose to secondary bacterial infections prompting treatment with antimicrobial agents [1]. The principal bacterial pathogens include *Mannheimia haemolytica* and *Pasteurella multocida*. Morbidity rates have been reported to be 75%, with mortality of 24.5–44.8% in calves [2] and between 50 and 70% in feed lots [3,4]. Economic losses from BRD range from USD 800–900 million annually in the USA alone [5], and treatment costs also exceed USD 54 million annually [6]. Antimicrobial therapy remains an important intervention and may consist of metaphylaxis control, which is the treatment of groups of high-risk animals to minimize disease onset, and for BRD is generally with a macrolide antimicrobial agent [7]. Treatment refers to therapy for infection and may be with any one of a number of approved veterinary antibiotics, including beta-lactam, fluoroquinolone, macrolide, or phenicol agents [8,9]. In human medicine, it is well recognized that prompt antimicrobial therapy (4 h after clinical presentation) in patients with pneumonia impacts morbidity and mortality [10].

The susceptibility or resistance of bacteria to antimicrobial agents is determined by an in vitro measurement called the minimal inhibitory concentration (MIC) which uses a bacterial inoculum of 10^5^ colony-forming units per milliliter (CFU/mL). An alternative measurement called mutant prevention concentration (MPC) defines the antimicrobial drug concentration blocking the growth of the least susceptible cells present in high-density (≥10^9^ CFUs) bacterial populations. High-density bacterial populations have been documented in clinical infections in humans, including pneumonia [11], other respiratory tract infections [12], meningitis [13,14] and urinary tract infections [15,16]. In cattle, McVey and colleagues showed bacterial densities of ≥10^8^ CFUs in experimentally induced respiratory infections in cattle [17]. High density bacterial populations are concerning as organisms with reduced susceptibility, requiring higher drug concentrations for inhibition of growth, may exist as subpopulations and not be detected by standardized susceptibility testing. Antimicrobial therapy with achievable drug concentrations that are insufficient to block the growth of the least susceptible cells allows for selective amplification of the resistant cells in the presence of the drug—regardless of the mechanism of reduced susceptibility [18]. Therapeutic drug concentrations preventing selective amplification of resistant bacterial cells reduce resistance selection from bacterial populations tested susceptible by MIC testing. Dagan and colleagues provided evidence that eradication of bacteria causing respiratory tract infections was necessary for clinical recovery and reducing resistance spread [19].

Pradofloxacin is a third-generation [20] enhanced-spectrum, dual-targeting fluoroquinolone approved for use in veterinary medicine [21] and has broad-spectrum activity against Gram-positive, Gram-negative [22], atypical bacteria [20,23,24] and also against anaerobic organisms [25]. It simultaneously targets DNA gyrase and topoisomerase IV in both Gram-negative and Gram-positive bacteria. These enzymes are critical for bacterial DNA regulation. Pradofloxacin has been argued to have a reduced likelihood for resistance selection based on low MPC values [21,26] and dual enzyme targeting. Previous investigations on companion animal pathogens [18,27,28] reported low MIC and MPC values for pradofloxacin, and we were interested in determining similar measurements for two primary BRD pathogens—*M. haemolytica* (MIC_90_ ≤ 0.016, MPC_90_ 0.063) and *P. multocida* (MIC_90_ ≤ 0.016, MPC_90_ 0.031) with pradofloxacin compared to ceftiofur, enrofloxacin, florfenicol, marbofloxacin, tilmicosin, tildipirosin, and tulathromycin. All strains tested had pradofloxacin MIC and MPC values below the susceptibility breakpoints established for MIC testing.

## 2. Materials and Methods

### 2.1. Bacterial Strains

Clinical isolates of *M. haemolytica* (n = 34) and *P. multocida* (n = 40) collected from field trials in the USA were used. Organism identification was by both matrix-assisted laser desorption ionization—time of flight (MALDI-TOF) (BioMerieux, St. Laurent, QC, Canada) and Vitek II (BioMerieux, St. Laurent, QC, Canada). Bacterial strains were cultured on tryptic soy agar containing 5% sheep red blood cells (BA) (Oxoid, Nepean, ON, Canada) for 18–24 h in O_2_ at 35–37 °C. For storage, single colonies were picked, transferred to skim milk, and stored frozen at −70 °C. Each isolate included in the study had to be susceptible to each agent based on recommended susceptibility MIC breakpoints [29].

### 2.2. Antimicrobial Compounds

Enrofloxacin and pradofloxacin were obtained in powder form from Bayer Animal Health (Elanco, Greenfield, IN, USA as of 2020) and prepared based on the manufacturers’ instructions. Ceftiofur (Zoetis, Kirkland, QC, Canada), florfenicol (Merck, Kirkland, QC, Canada), marbofloxacin (Vetoquinal, Lavaltrie, QC, Canada), tildipirosin (Merck, Kirkland, QC, Canada), tilmicosin, and tulathromycin (Zoetis, Kirkland, QC, Canada) were purchased in commercial form and prepared as per the manufacturer’s directions. Formulation excipients had no effect on MIC or MPC determination. Fresh stock solutions or samples stored at −70 °C were used for each experiment. 

### 2.3. MIC Testing

MIC testing followed the procedure recommended by the Clinical and Laboratory Standards Institute [30]. Thawed isolates were sub-cultured (×2) on BA plates and incubated for 18–24 h in O_2_ at 35–37 °C. In 96-well microdilution trays, Mueller–Hinton Broth (MHB) (Difco Laboratories, Detroit, MI, USA) containing 2-fold drug concentration increments was added. A 0.5 McFarland standard was diluted to a final inoculum of 5 × 10^5^ cfu/mL and added to the microtiter trays, incubated for 18–24 h at 35–37 °C in O_2_. The lowest drug concentration preventing visible bacterial growth was recorded as the MIC. Four American Type Culture Collection (ATCC) control strains—*Enterococcus faecalis* 29212, *Escherichia coli* 25922, *Staphylococcus aureus* 29213, and *Pseudomonas aeruginosa* 27853—were tested with each MIC assay to ensure the assays were within acceptable quality control performance ranges. 

### 2.4. MPC Testing

Five BA plates per strain were inoculated for confluent growth, incubated for 18–24 h at 35–37 °C in O_2_, and the next day, the complete contents of the inoculated plates were transferred to 100 mL of MHB and incubated (18–24 h at 35–37C in O_2_) [31,32]. Cultures were subsequently estimated to have concentrations of ≥3 × 10^9^ cfu/mL by spectrophotometric readings (600 nm) > 0.3 (Thermo Scientific Genesys 10s vis, Mississauga, ON, Canada) and by colony counts. Aliquots (100 µL) containing ≥10^9^ cfu were applied to BA plates containing antimicrobial agent at drug concentrations ranging from one dilution below the measured MIC to seven dilutions above the MIC. BA plates containing antimicrobial agents were used within 1 week of preparation. Inoculated plates were incubated (as described) for a total of 48 h, with examination for growth at 24 and 48 h. The lowest drug concentration preventing all visible growth (48 h) was the MPC. Each experiment included the four aforementioned ATCC control strains.

## 3. Results

The MIC and MPC data for the *M. haemolytica* strains tested against eight antimicrobial agents are shown in Table 1. The drug concentrations (µg/mL) inhibiting 50% (MIC_50_), 90% (MIC_90_) and 100% (MIC_100_), respectively, of the strains tested were as follows: ceftiofur ≤0.016, ≤0.016, 0.031; enrofloxacin ≤0.016, ≤0.016, 0.031; florfenicol 1, 2, 2; marbofloxacin ≤0.016, ≤0.016, 0.016; pradofloxacin ≤0.016, ≤0.016, 0.016; tildipirosin 0.5, 1, 1; tilmicosin 0.5, 8, 8; tulathromycin 0.5, 1, 2. The MPC_50_, MPC_90_, and MPC_100,_ respectively, were as follows: ceftiofur 0.25, 1, 1; enrofloxacin 0.125, 0.25, 0.5; florfenicol 2, 4, 4; marbofloxacin 0.063, 0.063, 0.125; pradofloxacin 0.031, 0.63, 0.063; tildipirosin 2, 4, 8; tilmicosin 16, ≥32, ≥32; tulathromycin 2, 4, 8.

Table 2 summarizes MIC and MPC data for the *P. multocida* strains tested. The MIC_50_, MIC_90_ and MIC_100_ values (µg/mL) were as follows, respectively: ceftiofur ≤0.016, ≤0.016, ≤0.016; enrofloxacin ≤0.016, ≤0.016, ≤0.016; florfenicol 0.5, 0.5, 0.5; marbofloxacin ≤0.016, 0.031, 0.031; pradofloxacin ≤0.016, ≤0.016, 0.031; tildipirosin 0.5, 2, 2; tilmicosin 2, 4, 8; tulathromycin 0.25, 0.5, 0.5. The MPC_50_, MPC_90,_ and MPC_100_ values were as follows, respectively: ceftiofur ≤0.125, 0.25, 0.5; enrofloxacin 0.063, 0.125, 0.125; florfenicol 1, 1, 1; marbofloxacin 0.063, 0.125, 0.25; pradofloxacin ≤0.016, 0.031, 0.063; tildipirosin 4, 4, 8; tilmicosin 8, 16, 32; tulathromycin 2, 2, 4.

The modal MICs against *M. haemolytica*/*P. multocida* strains were ≤0.016 for ceftiofur, enrofloxacin, marbofloxacin, and pradofloxacin; 0.1/0.5 µg/mL for florfenicol; and were 0.5 tildipirosin, 0.5/1 for tilmicosin, and 0.5/2 for tulathromycin.

The modal MPC value against the *M. haemolytica* strain was lowest for pradofloxacin at 0.031 µg/mL, followed by ceftiofur at 0.125 µg/mL, marbofloxacin at 0.063 µg/mL, enrofloxacin at 0.125 µg/mL, tildipirosin and tulathromycin at 2 µg/mL, and tilmicosin at 4 µg/mL. For all agents—except tilmicosin, MPC values were at or below the susceptibility breakpoints. 

For *M. haemolytica* and *P. multocida,* the C_max_/MIC_90_ values were 118.8 for enrofloxacin, 93.8 for marbofloxacin, and 212.5 for pradofloxacin. By comparison, C_max_/MPC_90_ values were 7.6, 23.8, and 53.9, respectively (Table 3).

## 4. Discussion

Fluoroquinolones are an important class of bactericidal antimicrobial agents for treating bacterial infections, including BRD, and pradofloxacin is the newest approved veterinary fluoroquinolones and has a number of favorable characteristics. Like other fluoroquinolones, it is active against Gram-positive and Gram-negative bacteria and against atypical bacteria and has favourable pharmacokinetic and pharmacodynamic properties. Pradofloxacin is also active against anaerobic bacteria [25].

Previously approved veterinary fluoroquinolones, including enrofloxacin, marbofloxacin, orbifloxacin, and difloxacin, preferentially target one of two enzymes (e.g., DNA gyrase or topoisomerase IV) critical for bacterial DNA replication. Typically, topoisomerase IV is the target in Gram-positive bacteria and DNA gyrase in Gram-negative bacteria. Pradofloxacin—as a dual-targeting fluoroquinolone—simultaneously inhibits both enzymes. Mutations in either of the *parC* or *gyrA* genes elevate MIC values and lessen the activity of single-target drugs. If the MIC is elevated above the susceptibility breakpoint, the organism is then considered non-susceptible or resistant. Pradofloxacin as a dual-targeting drug would require simultaneous mutations in both the *parC* and *gyrA* genes for resistance to occur.

The mutant prevention concentration (MPC) defines the antimicrobial drug concentration that blocks the growth of the less susceptible cells present in high-density bacterial populations [18]. MPC testing is similar to MIC testing, with some important differences: first, the inoculum (10^5^ cfu/mL—MIC versus ≥10^9^ CFUs MPC); second, MPC testing is currently conducted by agar dilution with antimicrobial compounds incorporated into the agar plates; and third, the MPC endpoint is 100% inhibition of bacterial growth. In this study, only organisms testing susceptible to each of the antimicrobials were included, as MPC testing is only performed on organisms testing susceptible to recommended breakpoints [18]. 

The MIC_50/90_ and MPC_50/90_ values for enrofloxacin, florfenicol, tilmicosin, and tulathromycin against the *M. haemolytica* isolates are similar to those previously published on a larger collection of strains [32]. Similarly, the MIC_50/90_ and MPC_50/90_ values for the same five drugs against the *P. multocida* strains in this study are similar to values previously reported for *P. multocida* strains from swine [38].

In this study, 100% of the strains tested had MPC values of ≤0.063 µg/mL—4-fold below the susceptibility breakpoint of ≤0.25 µg/mL for pradofloxacin. Fluoroquinolones are characterized as concentration-dependent agents, and their activity is based on the maximum serum-to-MIC ratio (C_max_/MIC) and the area of the drug concentration curve to MIC ratio (AUC/MIC). The exact values for the C_max_/MIC or AUC/MIC need to be debated; however, values of 10–12 and ≥125, respectively, have been used to show a more favorable clinical outcome and perhaps to minimize resistance selection. For pradofloxacin the C_max_/MIC ratio was 212.5 and the AUC/MIC was 825 for both organisms. 

The free fraction of the drug represents the unbound percentage [39] that is accepted to contribute to the antimicrobial effect of pradofloxacin at approximately 40% protein bound. The C_max_/MIC ratio would be 127.5 and the AUC/MIC would be 495—both ratios well above the aforementioned ratio considered important for minimizing resistance and a favorable clinical outcome.

MIC testing is universally accepted as the principal antibacterial measurement for PK/PD modeling. Zhang and colleagues commented on MIC testing and potential limitations [40]. Indeed, they suggested that MIC test methods may contribute to treatment failure and the emergence of resistant mutants due to the following variables: (1) MIC testing is an all-or-none measurement with antibacterial activity only when the drug concentration is at or exceeds the MIC, but, in fact, a lower drug concentration may exert some level of antibacterial activity; (2) MIC testing is by doubling dilutions, which may elevate drug concentration with prolonged residual time; (3) MIC measurements are with static drug concentration; and (4) bacterial cell densities are 10^5^ cfu/mL, which is well below the organism density that occurs during infection. Based on the above, the authors suggested that PK/PD modeling should also include MPC measurements, as reported here.

Ahmad and colleagues suggested MPC measurements need to be incorporated into PK/PD to assist with dosing guidelines [41]. In support of this argument, Xu et al. reported that Time>MPC was more important than T>MIC in the dosage design for preventing antimicrobial resistance for antimicrobial drug use (enrofloxacin) in aquaculture [42]. Cui and colleagues working with *Staphylococcus aureus* reported that AUC_0–24_/MPC above 25 h restricted the acquisition of resistance [43]. Olofsson et al. working with *E. coli* and ciprofloxacin in an in vitro kinetic model, reported that an AUC/MPC ratio of ≥22 was the single pharmacodynamic index that predicted the prevention of resistant mutant enrichment [44]. Vilalta et al. working with marbofloxacin, *Haemophilus parasuis*, and *Actinobacillus pleuropneumoniae*, indicated that marbofloxacin should prevent resistance selection for strains with MPC values up to 1 µg/mL [45]. Additionally, an AUC/MPC >25 h correlated with resistance prevention. Liang et al. investigated levofloxacin-resistant strains of *S. aureus*—same MIC but different MPC values—and compared differences between AUC_24_/MIC and AUC_24_/MPC for inhibiting the emergence of drug-resistant bacteria. Drug-resistant mutants were inhibited with AUC_24_/MPC values between 22 and 25. AUC_24_/MPC appeared more suitable than AUC_24_/MIC due to up to 8-fold differences for strains having the same MIC. Zhang and colleagues are investigating danofloxacin and *A. pleuropneumoniae* in a porcine tissue cage infection model showed the selection of drug-resistant bacteria could be significantly inhibited when the AUC_24h_/MPC was >18.58 h [46]. While the above investigations apply to concentration-dependent drugs, the application of time in the mutant selection window and T>MPC can be applied to time-dependent drugs. Alieva et al. investigated linezolid in an in vitro dynamic model [47]. They measured the drug residence time in the mutant selection window and the emergence of drug resistant bacteria. Time within the MSW was an important predictor for the selection and emergence of drug resistant bacteria. Xiong et al. showed drug-resistant bacteria occurred when T>MIC_99_ > 70% or T>MPC < 58% when studying cefquinome and *S. aureus* [48]. Zhang et al. investigating cefquinome and *E. coli*, showed selection and amplification of resistant bacteria when T>MIC_90_ > 25% or T>MPC was <50% [49]. The above-noted studies for both concentration and time-dependent agents showed MPC-based measurements integrated into PK/PD modeling for resistance prevention.

In considering MPC (versus MIC) values for pradofloxacin in C_max_ and AUC calculations based on the data from our study, the C_max_/MPC_90_ value was 53.9 for *M. haemolytica* and 109.7 for *P. multocida*. The AUC_24_/MPC_90_ values were 209.5 for *M. haemolytica* and 425.8 for *P. multocida*. Drug curves considering MIC_90_ and MPC_90_ and the MSW showed pradofloxacin would exceed these values for >12 and <18 h of the drug concentration curve, respectively. For *M. haemolytica*, the peak pradofloxacin drug concentration was 50× the MIC_90_ and 12× the MPC_90_. For *P. multocida*, those values were 50× and 26×, respectively. 

Fluoroquinolones are considered critically important antimicrobial agents in human health. The use of critically important compounds in food animals has been questioned; however, Kasimanickan et al. commented that there is no alternative to antimicrobials to treat non-vaccine preventable infectious diseases [50]. To this point, the authors argue that food animal producers need to play an important role in preventing overuse and misuse of antimicrobial agents. Scott and colleagues commented on the need for research on alternative therapies for infectious diseases in animals and for more judicious use of antibiotics in food animals. Label restrictions are intended to reduce inappropriate and non-approved use of drugs [51].

Pradofloxacin is the newest of the veterinary fluoroquinolones to be approved for use in food animals. The favorable in vitro activity against the principal BRD bacterial pathogens and favorable pharmacological characteristics—including dual targeting against critical enzymes for DNA replication—suggest an agent with a low likelihood for resistance selection against the pathogens tested.

## Figures and Tables

**Table 1:** MIC and MPC distribution values for 34 *M. haemolytica* strains.

Drug	Bacteriostatic(S)/ Bactericidal(C)	MIC/MPC Distribution Values (µg/mL)													
		≤0.016	0.031	0.063	0.125	0.25	0.5	1	2	4	8	16	≥32		
						MIC								MIC Breapoint	MIC_50/90/100_
Ceftiofur	C	33	1											≤2	≤0.016/≤0.016/0.031
Enrofloxacin	C	33	1											≤0.25	≤0.016/≤0.016/0.031
Florfenicol	S							23	11					≤2	1/2/2
Marbofloxacin	C	34												≤1 *	≤0.016/≤0.016/2
Pradofloxacin	C	34												≤0.125	≤0.016/≤0.016/≤0.016
Tildipirosin	S					2	19	13						≤4	0.5/1/1
Tilmicosin	S					2	18	2	1	2	9			≤8	0.5/8/8
Tulathromycin	S						22	11	1					≤16	0.5/1/2
MPC															MPC_50/90/100_
Ceftiofur				3	13	6	3	9							0.25/1/1
Enrofloxacin				7	15	10	2								0.125/0.25/0.5
Florfenicol								1	25	8					2/4/4
Marbofloxacin			1	32	1										0.063/0.063/0.125
Pradofloxacin		6	22	6											0.031/0.063/0.063
Tildipirosin									28	4	2				2/4/8
Tilmicosin										10	3	9	12		16/≥32/≥32
Tulathromycin									18	10	6				2/4/8

MIC = minimum inhibitory concentration; MPC = mutant prevention concentration. * For *Enterobacteriales.*

**Table 2:** MIC and MPC distribution values for 41 *P. multocida* strains.

Drug	Bacteriostatic(S)/ Bactericidal(C)	MIC/MPC Distribution Values (µg/mL)													
		≤0.016	0.031	0.063	0.125	0.25	0.5	1	2	4	8	16	≥32		
						MIC								MIC Breakpoint	MPC_50/90/100_
Ceftiofur	C	41												≤2	≤0.016/≤0.016/≤0.016
Enrofloxacin	C	41												≤0.25	≤0.016/≤0.016/≤0.016
Florfenicol	S					18	23							≤2	0.5/0.5/0.5
Marbofloxacin	C	36	5											≤1 *	≤0.016/0.031/0.031
Pradofloxacin	C	38	3											≤0.125	≤0.016/≤0.016/0.031
Tildipirosin	S			1	1	5	14	10	10					≤8	0.5/2/2
Tilmicosin	S						2	2	20	15	2			≤8 **	2/4/8
Tulathromycin	S				8	24	9							≤16	0.25/0.5/0.5
		MPC													MPC_50/90/100_
Ceftiofur		3	8	5	12	12	1								0.125/0.25/0.5
Enrofloxacin		4	11	15	11										0.063/0.125/0.125
Florfenicol							3	38							1/1/1
Marbofloxacin		4	10	13	12	2									0.063/0.125/0.25
Pradofloxacin		22	18	1											≤0.016/0.031/0.063
Tildipirosin								2	8	28	3				4/4/8
Tilmicosin									2	8	20	7	4		8/16/≥32
Tulathromycin								10	27	2	2				1/2/8

* For Enterobacteriales.** For *M. haemolytica*.

**Table 3:** Pharmacokinetic and pharmacodynamic values for eight antimicrobial agents.

Compound	C_max_	T_issuemax_	AUC_24_	C_max_/ MIC_90_	C_max_/ MPC_90_	AUC_24_/ MIC_90_	AUC_24_/ MPC_90_	T>MIC_90_	T>MPC_90_	% Protein Binding	Concentration (C) or Time (T) Dependent
***M. haemolytica***											
Ceftiofur *	6.9	2.64	376	431.3	6.9	23,500	376	10 days	4–5 days	95 **	T>MIC
Enrofloxacin	1.9	4.6	20.71	118.8	7.6	1294.4	80.7	>24 h	22 h	~46	AUC/MIC, C_MAX_/MIC
Florfenicol	3.7	2.94	101.9	1.85	0.93	50.9	25.5	24 h	2 h	~20	T>MIC
Marbofloxacin [33]	1.5		6.9	93.8	23.8	431.3	109.5	>24 h	~18 h	~30	AUC/MIC, C_MAX_/MIC
Pradofloxacin	3.4	0.81	13.2	212.5	53.9	825	209.5	>72 h	>24 h	~40	AUC/MIC, C_MAX_/MIC
Tildipirosin [34]	0.77	14.8	24.9	0.77	0.19	24.9	6.2	0	0	~30	AUC/MIC
Tilmicosin [35,36,37]	0.87		17.2	0.11	0.03	2.2	0.54	0	0	~25	T>MIC
Tulathromycin	0.6	3.2	63.7	0.6	0.08	63.7	15.9	0	0	~40	T>MIC
***P. multocida***											
Ceftiofur	6.9	2.64	376	431.3	27.6	23,500	1504	10 days	6 days		
Enrofloxacin	1.9	4.6	20.71	118.8	15.2	1294.4	165.7	>24 h	>24 h		
Florfenicol	3.7	2.94	101.9	7.4	3.7	203.8	101.9	96 h	>2 h		
Marbofloxacin	1.5		6.9	93.8	12	222.6	55.2	20 h	~10 h		
Pradofloxacin	3.4	0.81	13.2	212.5	109.7	825	425.8	>24 h	>12 <18 h		
Tildipirosin	0.77	14.8	24.9	0.38	0.19	12.5	6.2	0	0		
Tilmicosin	0.87		17.2	0.21	0.05	4.3	1.1	0	0		
Tulathromycin	0.6	3.2	63.7	1.2	0.3	127.4	31.9	0	0		

* Based on total drug and does not account for protein binding. ** For desfuroylceftiofur.

## Data Availability

The data that support the findings of this study are available from the corresponding author upon reasonable request.
